# HIV-1 Envelope Glycoproteins Induce the Production of TNF-α and IL-10 in Human Monocytes by Activating Calcium Pathway

**DOI:** 10.1038/s41598-018-35478-1

**Published:** 2018-11-21

**Authors:** Rémi Planès, Manutea Serrero, Kaoutar Leghmari, Lbachir BenMohamed, Elmostafa Bahraoui

**Affiliations:** 1 INSERM, U1043 Toulouse, France; 2 CNRS, U5282 Toulouse, France; 3 University Paul-Sabatier, Toulouse, France; 4 Laboratory of Cellular and Molecular Immunology, Gavin Herbert Eye Institute, University of California Irvine, School of Medicine, Irvine, CA 92697 United States of America

## Abstract

Human HIV-1 infection leads inevitably to a chronic hyper-immune-activation. However, the nature of the targeted receptors and the pathways involved remain to be fully elucidated. We demonstrate that X4-tropic gp120 induced the production of TNF-α and IL-10 by monocytes through activation of a cell membrane receptor, distinct from the CD4, CXCR4, and MR receptors. Gp120 failed to stimulate IL-10 and TNF-α production by monocytes in Ca^2+^ free medium. This failure was total for IL-10 and partial for TNF-α. However, IL-10 and TNF-α production was fully restored following the addition of exogenous calcium. Accordingly, addition of BAPTA-AM and cyclosporine-A, fully and partially inhibited IL-10 and TNF-α respectively. The PKA pathway was crucial for IL-10 production but only partially involved in gp120-induced TNF-α. The PLC pathway was partially and equivalently involved in gp120-induced TNF-α and IL-10. Moreover, the inhibition of PI3K, ERK1/2, p38 MAP-kinases and NF-κB pathways totally abolished the production of both cytokines. In conclusion, this study revealed the crucial calcium signaling pathway triggered by HIV-1 gp120 to control the production of these two cytokines: TNF-α and IL-10. The finding could help in the development of a new therapeutic strategy to alleviate the chronic hyper-immune-activation observed in HIV-1 infected patients.

## Introduction

HIV-1 primarily targets CD4 positive cells, including CD4+ T-lymphocytes, monocytes/macrophages and, to a lesser extent, dendritic cells^1,2^. This targeting is initiated by the interactions between HIV-1 envelope glycoproteins and CD4 receptor, followed by the recruitment of CCR5 or CXCR4 co-receptors depending on the viral tropism. Primo-infections are mediated by R5-tropic viruses^3^, which use the CCR5 co-receptor. R5/X4 dual-tropic and, to a lesser extent, pure X4-tropic viruses, which use CCR5 and/or CXCR4 co-receptors appear later, about 8 to 10 years after the primo-infection, in about 40 to 50% of HIV-1 patients, due to a shift/extension from the R5 to R5/X4 or X4 tropism^4–6^. The interaction between gp120 and signaling coupled receptors leads to the production of several cytokines/chemokines including pro-inflammatory TNF-α^7^ and anti-inflammatory IL-10, two proteins implicated in the immuno-pathology often observed in HIV-1 infected patients. Earlier studies have clearly shown that increased levels of TNF-α and IL-10 in the plasma is associated with the evolution of AIDS-diseases^8,9^. In agreement with this observation, blockade of the TNF-α pathway in HIV-1 infected patients, following an anti-TNF-α mAbs therapy, led to improvement of AIDS symptoms^10^. The beneficial anti-TNF-α therapy has been extensively explored in the SIV-239 pathogenic/macaque model^11^. Although anti-TNF-α mAbs treatment (adalimumab) did not reduce viral load, it clearly improved immunological status, as shown by: (*i*) a down-modulation of pro-inflammatory gene expression; (*ii*) a decrease in the infiltration of neutrophils into the para-cortical T-cell area; (*iii*) a decrease in the expression of immunosuppressive cytokines including IL-10 and TGF-β; (*iv*) a reduction in fibrosis of the lymphoid tissue; and (*v*) a better preservation of CD4+ T-cell counts^11^.

Besides TNF-α, the IL-10, a highly immunosuppressive cytokine, also seems to play a key role in HIV-associated immune dysregulation^12–14^. Previous studies have demonstrated that: *(i)* an increased level of IL-10 is positively correlated with increased viral load and progression to the symptomatic stage of the disease in HIV-1 progressors^12–14^; *(ii)* patients producing less IL-10, due to the mutation IL-10–5′–592A on the IL-10 promoter, evolve less rapidly to the AIDS stage^15^; *(iii)* elite controllers, who do not develop AIDS diseases despite their seropositive status, do not show an increase in IL-10^16^; and *(iv)* in patients receiving effective anti-retroviral therapy, plasmatic IL-10 level decreases in parallel with the decrease in viral load^16^. In line with these observations, *in*-*vitro* studies have shown that IL-10 is directly involved in the loss of immune system function and immune-pathology. Clerici *et al*.^8^ and, more recently, Brockman *et al*.^16^ have shown that neutralization of the IL-10 pathway using antibodies blocking the IL-10-IL-10R interaction, allows immune functions to be restored in HIV-positive patients. Among the beneficial effects of this treatment, the authors observed a restoration of the proliferation of HIV-1 specific CD4+ and CD8+ T cells as well as increased production of IFN-γ and IL-2 cytokines. Interestingly, in another model of persistent viral infection of mice by LCMV clone 13, such a treatment was reported to lead to viral clearance^17^. Thus IL-10 appears to play a major role in the viral escape from the immune system. Accordingly, several HIV-viral proteins can induce the production of IL-10, including Tat^18–20^, Nef^21^ and gp120^22^. It remains therefore to determine the molecular mechanisms involved in IL-10 production during the course of HIV-1 infection. In addition, it is important to note that the stimulation of the expression of IL-10, and also of other immunosuppressive factors such as PD-L1, TIM3 and IDO, are preceded by the establishment of a chronic production of IFN-I^23^. Several reports have demonstrated the importance of the blockade of interferon type-I signaling in the control of immune activation, co-inhibitory immunosuppressive markers and persistent viral infection as observed in HIV-1, SIV and LCMV infections^24–26^.

Cytokines production in HIV-1 infected patients is the result of activation of several signaling pathways mediated by the interaction of various HIV-1 viral components, including gp120, with various cellular receptors, including CD4^7^ and CCR5. The several signaling pathways reported to be activated following gp120-CD4-CCR5/CXCR4 interactions include the activation of P56lck, a tyrosine kinase of the src family, associated with an intracytoplasmic domain of CD4^27^. This lead to the activation of downstream cascade signaling pathways, including elevation of intracellular mobilization of the calcium and the related nuclear factor of activated T-cells (NFAT) transcription factor^28,29^, activation of phospholipase C, PKC^30^, PyK2^31^, PI3K^32,33^, Akt^34^, Rac^35^; and MAP kinases ERK1/2^36^. Interestingly, the engagement of CXCR4 by gp120 also activates the downstream targets of the Rho family GTPases including LIM kinase^37^ and cofilin^38^, two factors directly implicated in the actin cytoskeleton rearrangement, a step that seems to be important to overcome the barrier of cortical actin in quiescent CD4+ T cells.

In addition to mediate productive viral infection following interaction with CD4, CCR5 and CXCR4, the gp120 also bind to several other receptors expressed by non-permissive cells including integrin α4β7^39^, C-type lectin DC-SIGN^40^, the mannose receptor MR^22^, TLR2, and TLR4^41^. Activation of these receptors is mediated by viral particles, either infectious or not, viral proteins expressed on infected cells, or soluble envelope glycoproteins that are secreted or released following lysis of infected cells. Also noteworthy is the multiple variety of interactions between the receptors of innate immune system on one hand and the viral protein and nucleic acids on the other hand, including TLR2^42^, TLR4^42,43^, TLR7/8^44^, cGAS^45^, and IFI16^46^. These interactions have been reported to be involved in the establishment of persistent chronic hyper-activation of the immune system. This hyper-immune-activation was associated with the impairment of several immune system components and functions, including a gradual depletion of CD4+ T-cells^47,48^, increased T-cell exhaustion^49^, decrease in the number of plasmacytoid and myeloid DCs^50,51^ and increased production of immune-modulatory cytokines/chemokines and costimulatory factors^52,53^.

Because of the importance of TNF-α and IL-10 cytokines in the immuno-pathology of HIV-1 infection, several studies have focused on understanding the signaling pathways and the receptors engaged by gp120 R5-tropic on both permissive and non-permissive cells^34,54^. However, the nature of receptors activated by gp120 from X4-tropic viruses and the subsequent signaling pathways triggered following this interaction remain to be fully elucidated. In the present study, we demonstrate that the HIV-1 gp120 stimulated the production of both TNF-α and IL-10 in human monocytes, in a CD4 and CCR5/CXCR4 independent manner, by recruiting selective, specific and common pathways. Moreover, we demonstrate that calcium and PKA pathways are essential for IL-10, but not for TNF-α production by monocytes stimulated by gp120, while PI3K, ERK1/2 and P38 MAP kinases, and NF-κB are essential for the production of both TNF-α and IL-10.

## Materials and Methods

### Ethics statement

The use of human cells in this study was approved by the Research Ethical Committee of Haute-Garonne, France. Human Peripheral Blood Mononuclear Cells (PBMC) were isolated from buffy coat of healthy human donors. Buffy coats were provided anonymously by the EFS (Etablissement Français du Sang, Toulouse, France). Written informed consent was obtained from the donors under EFS contract N° 21/PVNT/TOU/INSERM01/2011-0059, according to French Decree N° 2007–1220 (articles L1243-4, R1243-61).

### Isolation of human monocytes

PBMCs were isolated by centrifugation using standard Ficoll-Paque density (GE Healthcare). Briefly, the blood was diluted 1:1 in phosphate-buffered saline (PBS) pre-warmed at 37 °C and carefully layered over the Ficoll-Paque gradient. The tubes were centrifuged for 25 minutes at 2000 rpm at 20 °C. The cell interface layer was harvested carefully, and the cells were washed twice in PBS (for 10 minutes at 1200 rpm followed by 10 minutes at 800 rpm) and re-suspended in RPMI-1640 supplemented with 10% of foetal calf serum (FCS), 1% penicillin (100 IU/mL) and streptomycin (100 µg/ml). Monocytes were separated from lymphocytes by adherence to tissue culture plastic (Beckton Dickinson). PBMC were seeded in 24-well plates (10^7^ PBMC/well). After incubation for 1 hour at 37 °C, non-adherent cells were removed by 3 washes with PBS and adherent cells represented the monocyte fraction of PBMC (>94% CD14+, characterized by FACS).

### Recombinant proteins and peptides

Recombinant CXCR4 tropic HIV-1 gp120_Lai_ protein was obtained from the Agence Nationale de la Recherche sur le SIDA (Paris, France) and purified from the supernatant of BHK cells infected with vaccinia virus recombinant for gp-120. X4 gp120_HXB2_ was obtained from the NIBSC program, while X4 gp120_Baculo_ was produced and purified by our group. R5 gp120_Bal_ and R5 gp120_YU2_ were obtained from the NIH AIDS Reagent Program. Recombinant proteins are in a monomeric structures. HeLa-gp_Lai_ and HeLa-gp_Ada_, stably expressing gp120 Lai or Ada respectively and gp41 were used as sources of native like X4 and R5-tropic cell surface envelope glycoproteins. HIV-1-infected Jurkat cell lines were used in co-culture as sources of free HIV-1 particles and cells expressing viral proteins. Soluble recombinant CD4 was a gift from Professor David Klatzmann^55^. The anti-fusion synthetic peptide C34L, sequence 628–661 of HIV-1gp41_Lai_, was synthesized chemically.

### Antibodies and chemical inhibitors

mAb Leu3A was purchased from BD Biosciences. mAb 2G12 was obtained from the NIH AIDS Reagent Program. The following inhibitors were purchased: AMD300 (NIH AIDS Reagent Program); LPS (InvivoGen); cycloheximide, cyclosporine A, PHA, and BAPTA-AM (Sigma Aldrich); U73122, H89, Ro31, Ly294002, PD98059, U0126, SB202190, and Bay11702 (Calbiochem).

### Cytokine quantification by ELISA

Cytokine quantification of TNF-α and IL-10 was performed using a specific ELISA kit (eBioscience). Briefly, the first monoclonal antibody was used for capture overnight at 4 °C. After three washes with PBS containing 0.05% Tween 20 (wash buffer), plates were saturated by adding 250 µl of a protein solution (diluent assay) for one hour at room temperature. After three washes, culture supernatants (100 µl/well) were added and incubated for 2 hours at room temperature. Plates were then washed three times and incubated for 1 hour at room temperature with a biotinylated anti-cytokine antibody. After five washes, the bound biotinylated antibody was detected by additional 30 minutes incubation with streptavidin peroxidase. After seven washes, plates were incubated with the enzyme substrate (TMB). The reaction was stopped by adding 50 µl of H_2_SO_4_ (4 N) to each well. Absorbance was read at 450 nm with a wavelength correction at 570 nm. Cytokines were quantified from a standard curve generated by using various concentrations of recombinant protein of each cytokine. The limit of detection of the cytokines was 4 pg/ml for TNF-α and 2 pg/ml for IL-10.

### Western blot analysis

Equal amounts of proteins (10–40 μg) were subjected to 10% SDS-PAGE and the separated proteins were transferred to a nitrocellulose membrane. The membrane was blocked with 5% of non-fat milk in Tris-buffered saline with 0.05% Tween 20 (TTBS) for 1 h, then washed with TTBS, and incubated with the primary antibody overnight at 4 °C. Immunoreactive bands were detected by incubation for 1 h with the appropriate anti-primary antibodies conjugated with horseradish peroxidase (DAKO). Proteins of interest were visualized using a chemiluminescent substrate ECL (Pierce, Rockford, IL).

### Signal transduction experiments

Monocytes were incubated for 30 minutes with/or without various signal transduction pathway inhibitors before HIV-1 gp120 (10 nM) was added. After 24 hours, cell supernatants were harvested and quantified by ELISA for the presence of TNF-α and IL-10. The remaining cells were analyzed for the toxicity of each inhibitor by trypan blue exclusion assay. Only inhibitors at concentrations found to be non-toxic by this assay were used in the present study.

### Statistical analyses

Statistical analysis was performed using GraphPad Prism software. All results were expressed as means +/− SD. All experiments were performed for a minimum of three times. Differences in the means for the different groups were tested using a Student’s t test, a one-way ANOVA followed by a Bonferroni post hoc test, or a two-way ANOVA followed by a Bonferroni post hoc test (as indicated in figure legends). P values < 0.05 were considered to be statistically significant.

## Results

### HIV-gp120 induces IL-10 and TNF-α production by human monocytes

**Figure 1:**
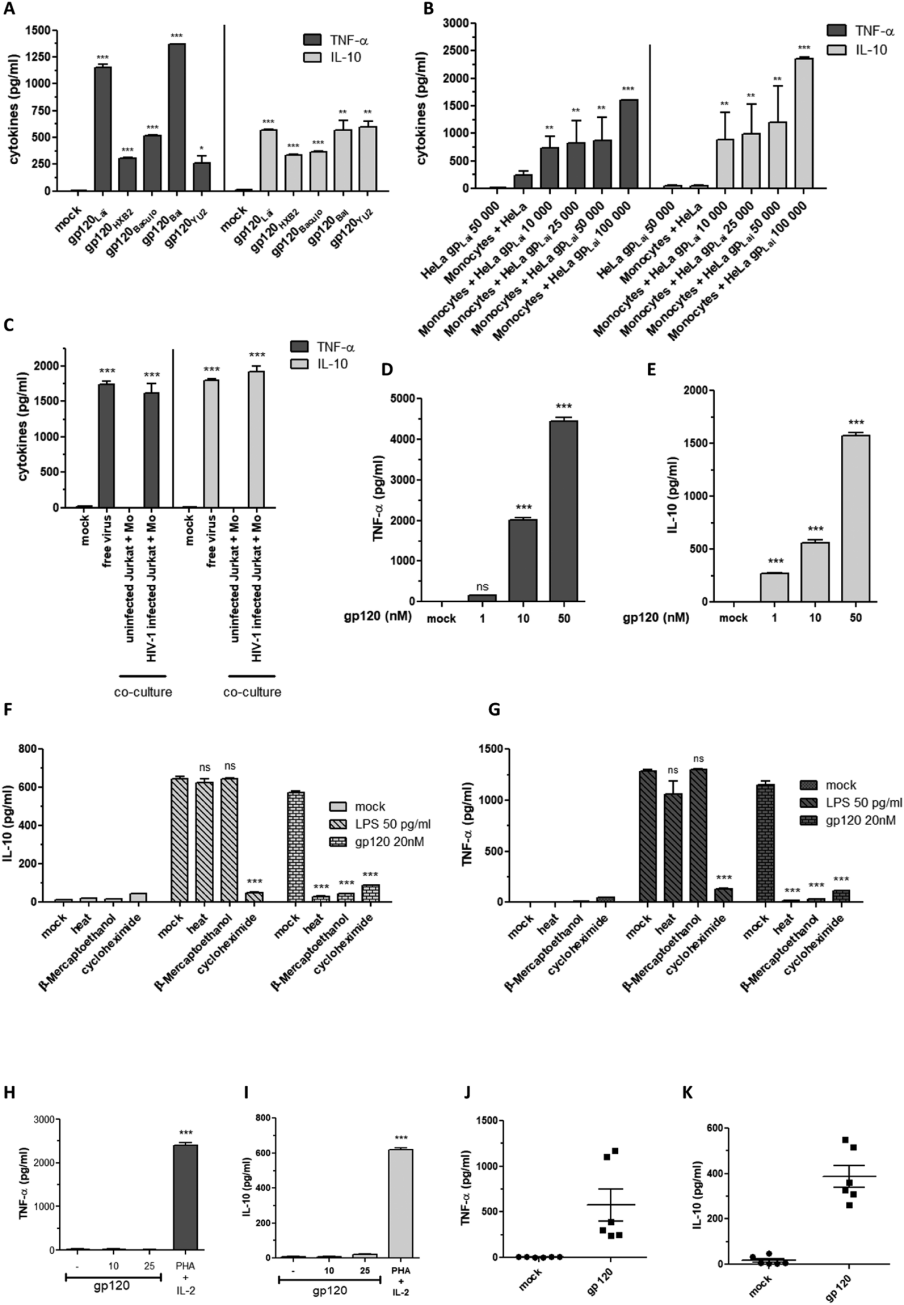
CCR5 and CXCR4 HIV-1 envelope glycoproteins induce the production of TNF-α and IL-10 in human monocytes. (**A**): Monocytes were mock treated or treated by X4-tropic HIV-1 gp120_Lai_, gp120_HXB2_, gp120_Baculo_, or R5-tropic gp120_Bal_, gp120_YU2_. Statistical significances were compared to “mock treated” group. (**B**) Monocytes were co-cultured with HeLa cells expressing X4-tropic HIV-1 gp120_Lai_ for 24 hours before cytokine quantification in the cell supernatants. As negative control monocytes were co-cultured with HeLa cells not expressing HIV-1 gp120. Statistical significances were compared to “monocytes + HeLa” group. (**C**) Effect of free HIV-1 Lai (50TCID50) or HIV-1 infected cells in stimulating the production of TNF-α and IL-10 in human monocytes. Statistical significances were compared to “mock” group. (**D**,**E**) Monocytes were treated for 24 h with gp120 (1 nM to 50 nM) and cytokines were quantified in the cell supernatants. Statistical significances were compared to “mock”-treated group. (**F**,**G**) Effect of gp120 denaturation, disulfide bridge reduction, or cycloheximide treatment on cytokine production by gp120 treated monocytes. Comparative similar experiments were performed with LPS. Statistical significances were compared to “mock” -treated group. (**H**,**I**) PBMC were depleted of monocytes by three successive adherence steps. Cells remaining in suspension (10^6^) were incubated in the presence of gp120 or treated by a mixture of PHA (3 µ/ml) and IL-2 (10 U/ml). The production of TNF-α and IL-10 in cells supernatants was determined by ELISA. Statistical significances were compared to untreated cells. (**J**,**K**) Monocytes from healthy human donors (n = 6) were treated with gp120 (10 nM), or not (Mock). After 24 h of treatment, TNF-α and IL-10 were quantified in the cell supernatants. Differences in the means for the different groups were tested using Student’s t test. Asterisks represent P values: *P < 0.05; **P < 0.01; ***P < 0.001.

Human monocytes, isolated from healthy donors, were stimulated by HIV–1 envelope glycoproteins from various sources including soluble gp120 produced in either vaccinia virus or baculovirus expression systems, cell surface expressed gp120/gp41 envelope glycoproteins, or viral free particles. After 24 hours, the production of TNF–α and IL–10 were quantified in cell supernatants by ELISA. The results showed that HIV–1 envelope glycoproteins, whatever the tropism R5 or X4, origin, or mode of presentation, induced TNF–α and IL–10 production (Fig. 1A–C).

Considering that several reports have been devoted to the R5–tropic envelope glycoproteins, in the rest of this study, we focused on characterizing the signaling pathways triggered by X4 gp120 to activate the production of TNF–α and IL–10 in human monocytes. To this end, we showed that treatment of monocytes with escalating amounts of HIV–1 gp120 (1, 10, or 50 nM) induced dose-dependent production of TNF-α and IL-10 (Fig. 1D,E). In control experiments, no significant productions of TNF-α or IL-10 were detected in the supernatants of untreated monocytes. To further characterize the specificity of this response and to demonstrate that the induction of TNF-α and IL-10 are not related to contaminant endotoxins but are directly related to the intrinsic effect of HIV-1 envelope glycoprotein, we showed that the production of these cytokines was totally abrogated when monocytes were stimulated with gp120 previously denatured by heating or following the reduction of disulfide bonds by β-mercaptoethanol treatment (Fig. 1F,G). As controls, we showed that similar treatment of LPS had no effect on its capacity to induce cytokine production as depicted in Fig. 1F,G. To investigate whether the secretion of TNF-α and IL-10 came from *de-novo* protein synthesis or from the mobilization of intracellular or membrane stocks, monocyte cells were stimulated with gp120 in the presence of cycloheximide (an inhibitor of protein synthesis). In these conditions, gp120-induced TNF-α and IL-10 production by monocytes was totally blocked, thus demonstrating that TNF-α and IL-10 were not released from intracellular/membrane stocks but were dependent on *de-novo* protein synthesis (Fig. 1F-G).

In order to identify the cell types that are able to secrete TNF-α and IL-10 in response to gp120 treatment, we split PBMCs into two distinct populations: plastic-adherent cells (mostly monocytes) and the plastic non-adherent population (mostly lymphocyte cells). After the first adherence step, which isolated mainly monocyte cells, the non-adherent cell fraction was subjected to three successive adherence steps and remaining non-adherent cells were stimulated by gp120 protein for 24 hours. The results showed that the non-adherent cell fraction did not produce TNF-α and IL-10 following treatment with 10 or 25 nM of gp120, while stimulation with phytohaemagglutinin (3 µg/ml) and IL-2 (10 U/ml), the two lymphocyte activators, led to a production of IL-10 and TNF-α. These results demonstrate that these two cytokines are rather selectively produced by the adherent cell fraction that contains the monocyte population (Fig. 1H,I). To generalize this observation, we showed that gp120 induced the production of TNF-α and IL-10 by monocytes isolated from six healthy donors (Fig. 1J,K). However, the amount of TNF-α and IL-10 secretion showed some variability between different donors, which may be related to genetic and/or epigenetic factors of the individual donor.

### Gp120 induces TNF-α and IL-10 production independently of CD4, CXCR4, MR interaction and viral entry

**Figure 2:**
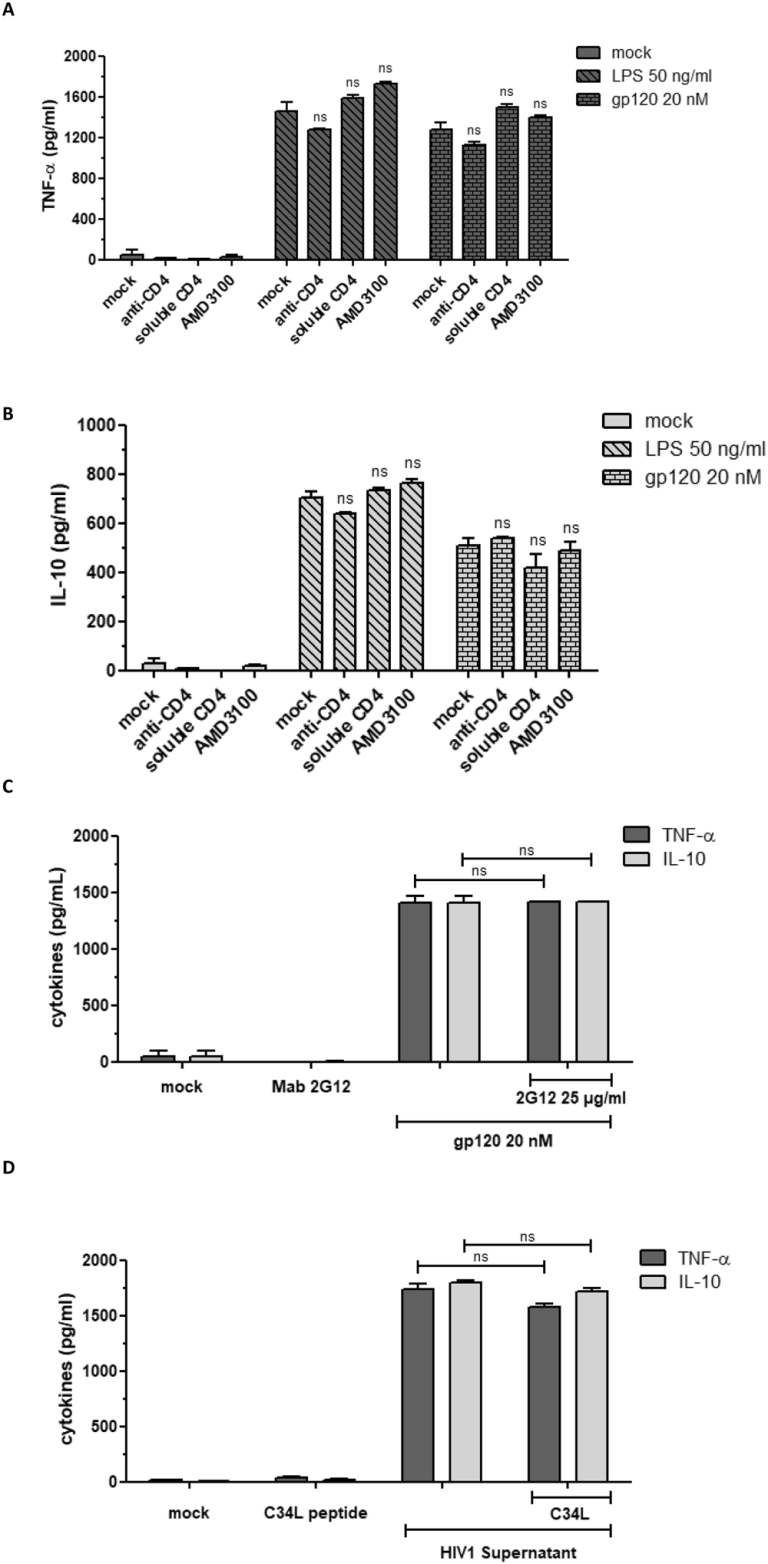
Activation of human monocytes by gp120 is CD4, CXCR4 and MR independent. (**A,B**) Monocytes (0.5 × 10^6^ cells) were “Mock” treated or previously treated for 1 hour at 37 °C with Leu3a anti-CD4 (2 µg/ml), soluble CD4 (5 µg/ml) or AMD300 (500 nM) before treatment with gp120 (20 nM) or LPS (50 ng/ml). After 24 hours, cell supernatants were harvested and the production of TNF-α and IL-10 was quantified by ELISA. (**C**,**D**) As above, monocytes were untreated (mock), pre-incubated for one hour with mAb 2G12 (25 µg/ml) or with anti-fusion peptide C34L (10^−6^ M) before treatment with gp120 (20 nM) (**C**) or with HIV-1 supernatant (**D**). Monocytes incubated with the mAb 2G12 or with the C34L peptide alone were used as negative controls. Statistical significances were compared to gp120 treated or HIV-1 supernatant treated cells.

To investigate the role of CD4 receptor and CXCR4 co-receptor in gp120-induced TNF-α and IL-10 production, monocytes were incubated with either the anti-CD4 neutralizing antibody Leu3a (2 µg/ml), soluble CD4 (5 µg/ml), or 500 nM of AMD3100, an antagonist of CXCR4 known for its ability to block the replication of X4-tropic HIV-1 isolates. In these conditions, no significant inhibition of TNF-α or IL-10 production was observed in response to gp120 treatment (Fig. 2A,B), suggesting that gp120-induced TNF-α and IL-10 occurred independently of the recruitment of receptor and co-receptor related signaling pathways. We further showed that gp120-induced cytokines were not blocked in the presence of *(i)* the broadly neutralizing 2G12 mAb, which recognizes a mannose carbohydrate epitope involved in the interaction with a previously described mannose receptor^22^ (Fig. 2C); *(ii)* the anti-fusion peptide C34L (10^−6^ M) (Fig. 2D); that efficiently inhibits viral entry^56^; these results suggest that virus entry is not essential for the induction TNF-α and IL-10 production.

Altogether, these results show that gp120-induced TNF-α and IL-10 production occurs independently of interaction with CD4, CXCR4 and mannose C type lectin receptors (MR), and does not require viral entry.

### Role of calcium, PLC, PKC and PKA pathways

Downstream of the phospholipase C (PLC), synthesis of inositol-1, 4, 5-triphosphate (IP3) induces an increase of cytoplasmic calcium mobilization responsible for activation of the calcium pathway. This calcium-mobilization leads to the activation of NFAT transcription factor following its dephosphorylation by calcineurin, an enzyme with a phosphatase activity. After activation, NFAT translocates to the nucleus, where it binds to cognate DNA sequences leading to transcriptional up-regulation of several genes including cytokines.

**Figure 3:**
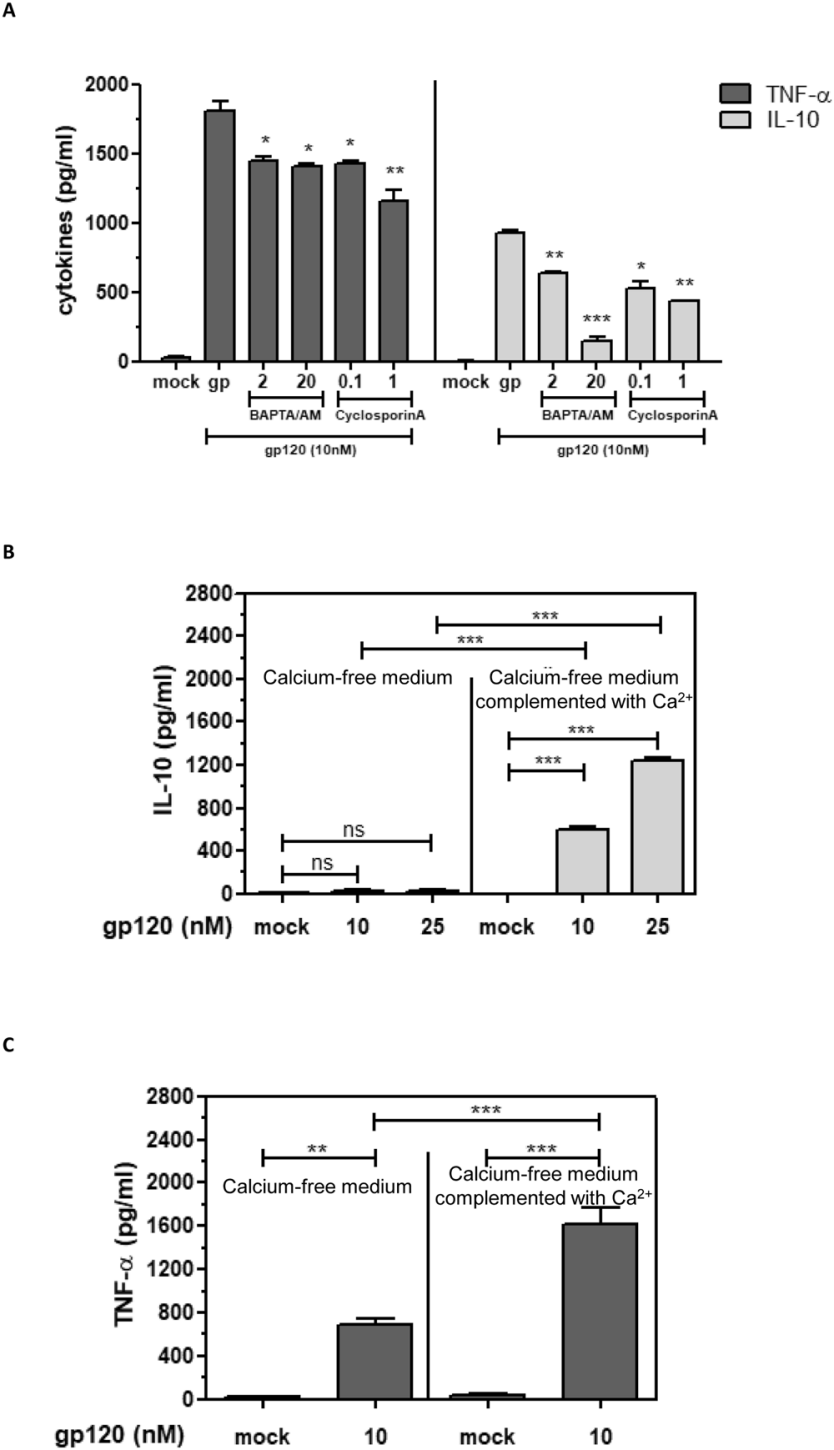
Calcium pathway is essential for gp120-induced IL-10 and strongly involved in TNF-α production. (**A**) Monocytes were pre-treated with BAPTA-AM at 2 or 20 µM or with cyclosporine-A before stimulation with gp120. After 24 hours, secreted cytokines were quantified by ELISA. Statistical significances were compared to gp120 treated cells in the absence of inhibitor. (**B**,**C**) Monocytes were incubated in a calcium-free medium (left) or in calcium-free medium complemented with 4 mM calcium chloride (right) and stimulated by gp120 (10–25 nM). Culture supernatants were then collected and the presence of TNF-α and IL-10 was determined by ELISA. The results are expressed in pg/ml of cytokines. The values represent means +/− SD of three independent experiments.

Previous reports have shown that gp120 is able to induce calcium mobilization in human macrophages^57^ and in U87 cell line stably transfected with HIV-1 CD4 receptor and CCR5 co-receptor^34^. Thus, we investigated whether the calcium pathway was involved in gp120-mediated IL-10 and TNF-α induction. Pre-incubation of monocytes with increasing concentrations of BAPTA/AM, an intracellular chelator of calcium, strongly inhibited gp120-mediated IL-10 production in a dose-dependent manner. Inhibition reached 80% when BAPTA/AM was used at 20 µM (Fig. 3A). Involvement of this pathway was also investigated using cyclosporine-A, an inhibitor of calcineurin. In the presence of cyclosporine-A, gp120 mediated IL-10 induction was inhibited by 45%. In order to further evaluate the crucial role of calcium in IL-10 induction, monocytes were stimulated by gp120 in a calcium-free medium. In the absence of calcium, gp120 used at 10 or 25 nM became unable to induce IL-10 (Fig. 3B). Interestingly, IL-10 production was restored if the calcium-free medium was complemented with exogenous calcium (CaCl_2_: 4 mM) (Fig. 3B). These results strengthen the conclusion that the role of calcium is crucial in the signaling pathways leading to the induction of IL-10 by HIV-1 gp120. In contrast to IL-10 production, TNF-α was only weakly inhibited by the stimulation of monocytes by gp120 (10 nM) in the presence of BAPTA/AM 20 µM and cyclosporine-A 1 µM, inhibition reaching 23% and 36% respectively. Furthermore, stimulation of monocytes with gp120 (10 nM) in a calcium-free medium still led to a significant production, about 42%, of TNF-α (Fig. 3C). More interestingly, the addition of calcium to the calcium-free medium rescued the total production of TNF-α. Thus gp120-mediated TNF-α was only partially dependent on the calcium pathway. Altogether, our data suggest that gp120 induced IL-10 via a strictly calcium dependent pathway, while induction of TNF-α seems to have been only partially calcium dependent. The latter data also suggest the implication of at least a second calcium-independent pathway in the gp120-induced TNF-α.

The role of calcium pathway may be related to the consequences of the activation of phospholipase C. Once activated, PLC cleaves phosphatidyl-inositol-phosphate (PIP2) into IP3 and diacylglycerol (DAG), which are responsible for the release of intracellular stocks of calcium and the activation of PKC respectively. Both pathways are known to be involved in cytokine gene expression. The activation of PLC and, downstream, PKC and calcium mobilization are also linked to the activation of PKA: a pathway recruited after elevation of c-AMP concentrations.

### Role of Phospholipase C

**Figure 4:**
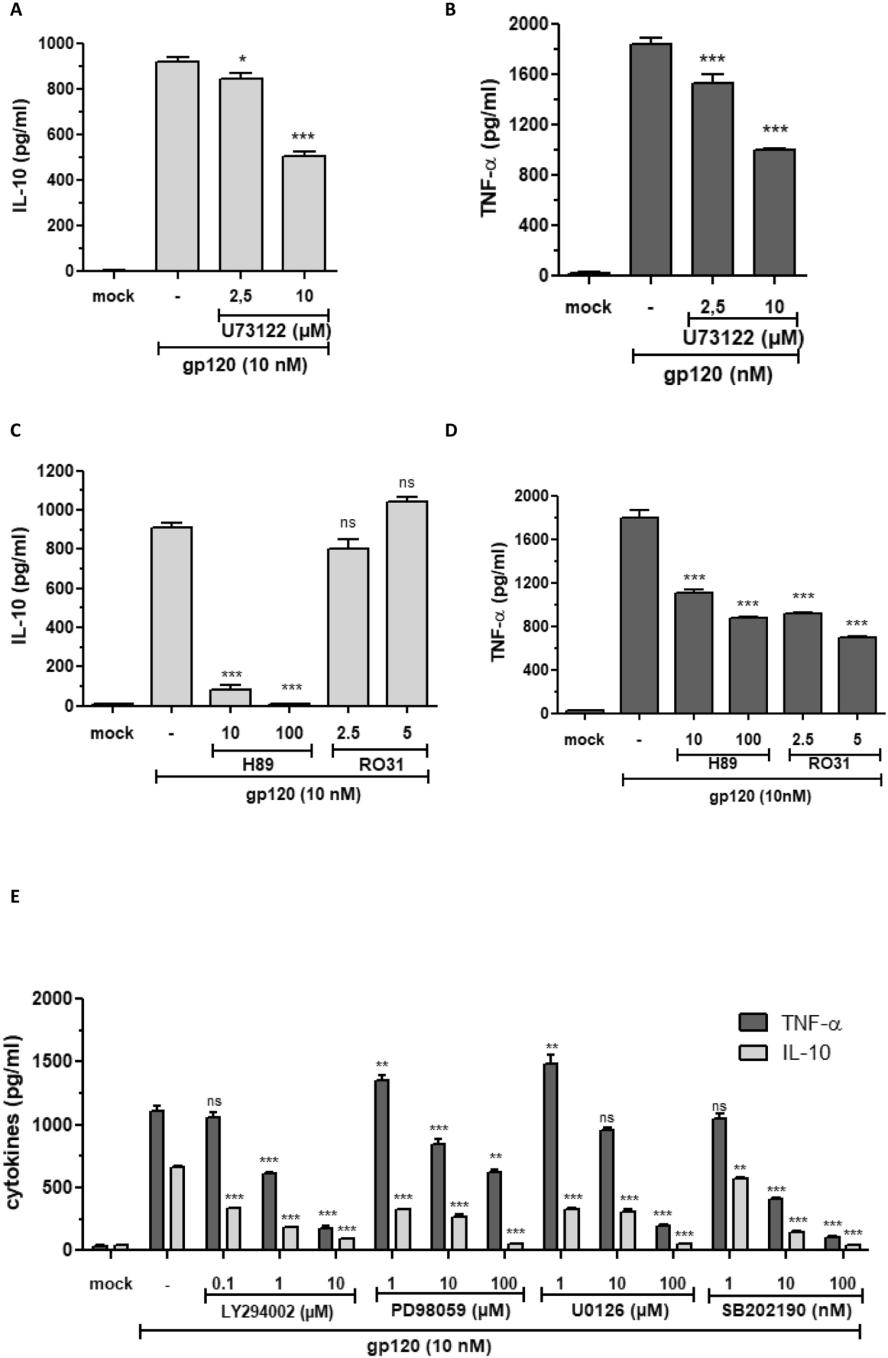
Effect of PLC, PKA, PKC, PI3K and MAP kinases pathway inhibitors on gp120-induced TNF-α and IL-10 cytokines. Before adding HIV-1 gp120 (10 nM), monocytes were either incubated or not for 30 minutes with different signal transduction pathway inhibitors: (**A**,**B**) U73122, an inhibitor of PLC; (**C**,**D**) H89 and RO31, inhibitors of PKA and PKC respectively; (**E**) Ly294002, inhibitor of PI3K, PD98059 and U0126, inhibitors of ERK1/2 MAP kinases, SB202190, inhibitor of P38 MAP kinase. After 24 h, cell supernatants were harvested and quantified by ELISA for the presence of TNF-α and IL-10. Statistical significances were compared to gp120 treated cells in the absence of inhibitor.

The role of PLC pathway in the control of the gp120-induced TNF-α and IL-10 production was evaluated using the chemical inhibitors PLC, U73 122. Briefly, monocytes were pre-incubated for 30 min with 2.5 and 10 µM of U73 122 before stimulation with gp120 at 10 nM. At 24 hours after treatment, cell supernatants were collected and cytokine production was quantified by ELISA. The results showed that U73 122 partially inhibited, but in a dose-dependent manner, both gp120-induced IL-10 and TNF-α production by 47% and 45% respectively (Fig. 4A, B). These results indicate that the PLC pathway seems to be involved in about half of the TNF-α and IL-10 production in response to gp120.

### Role of PKA and PKC

We then investigated the potential role of PKA and PKC, two protein kinases known for their involvement in the signaling pathways controlling expression of several cytokines. The PKA pathway is activated after elevation of c-AMP concentrations. The PKC pathway is activated by calcium and DAG for the classic PKC family (α, β1, β2, and γ isoforms) or by DAG only for non-classic PKC (δ, ε, η, θ and μ isoforms) while the activation of the atypical PKC family (ζ and υ/λ isoforms) is independent of Ca^2+^ and DAG, two components generated following the enzymatic action of PLC on PIP2. The role of PKA and PKC pathways downstream of PLC in gp120-induced TNF-α and IL-10 production was evaluated using the chemical inhibitors H89, an inhibitor of PKA, and Ro31–8220, an inhibitor of PKC. Strong and total inhibition of IL-10 production was observed in the presence of PKA inhibitor, while only a partial inhibition, of 39% and 55%, of TNF-α was obtained by H89 at 10 µM and 100 µM, respectively (Fig. 4C, D). In the presence of the PKC inhibitor Ro31–8220 at 2.5 and 5 µM, we observed strong inhibition of TNF-α of 51% and 61%, respectively, whereas IL-10 production was not affected (Fig. 4C). These data show that, downstream of PLC activation, selective pathways are involved in gp120-induced TNF-α and IL-10 production. While TNF-α production is partially dependent on calcium, PKA and PKC pathways, IL-10 production is completely dependent on calcium and PKA pathway but independent of PKC.

### Role of PI3K, MAP kinases and NF-κB pathways in gp120-induced TNF-α and IL-10 production

Because MAP kinases and PI3K are activated downstream of PLC pathway, we investigated their role in the control of gp120-induced TNF-α and IL-10 production. Briefly, human monocytes were pre-treated with chemical inhibitors targeting PI3K (Ly299002), MAPkinases ERK1/2 (PD98059 or U0126) or P38 (SB202190) and then stimulated with gp120 (10 nM). We found that inhibition of PI3K, MAPkinases ERK1/2 and p38 strongly inhibited, at least at the highest concentrations, gp120-induced TNF-α and IL-10 production (Fig. 4E). These results are in agreement with previously published data and confirmed the major role of these signaling pathways in the control of gp120-induced cytokines.

**Figure 5:**
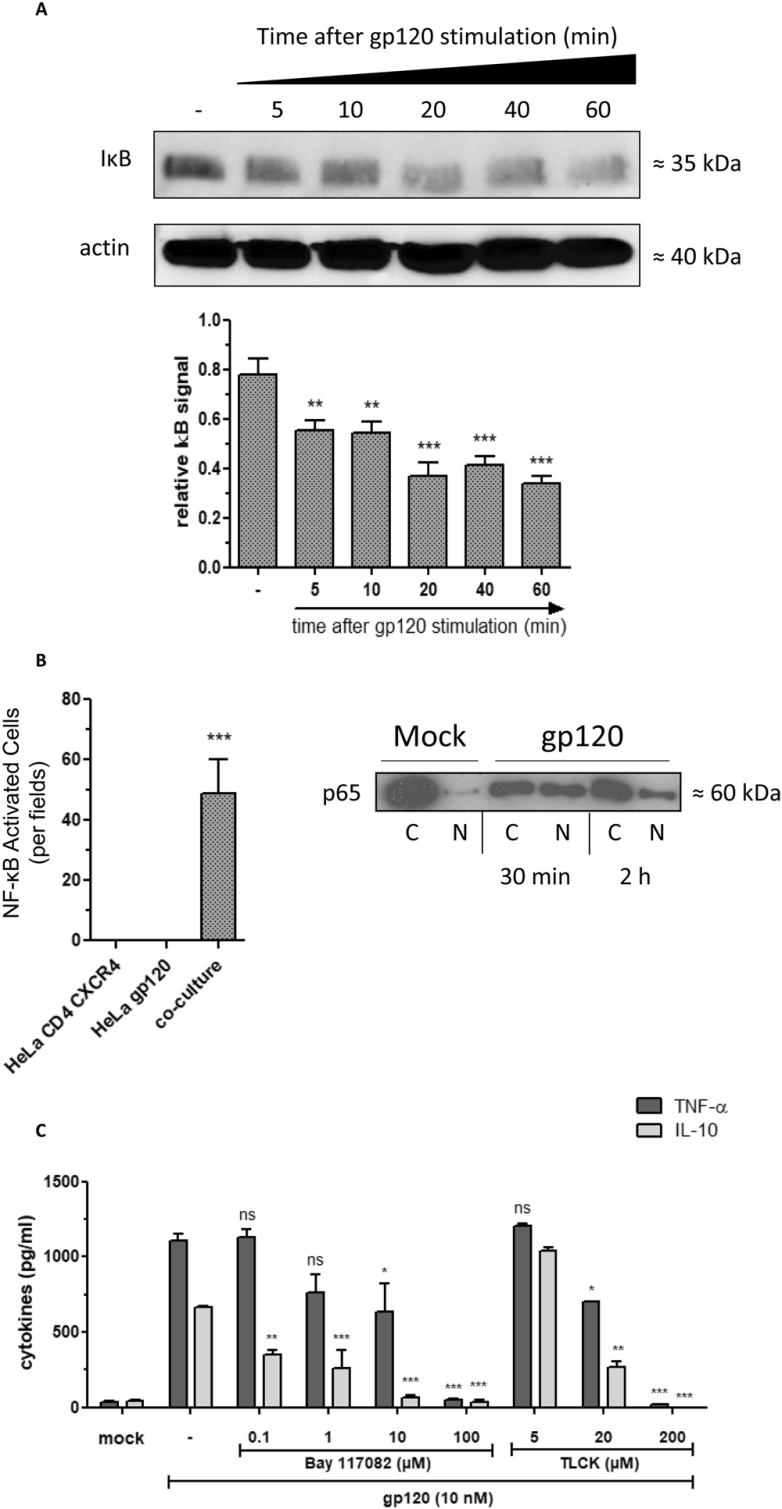
gp120-induced NF-κB is essential for the production of TNF-α and IL-10. (**A**) monocytes (3 × 10^6^) were stimulated by X4-gp120 during 5 to 60 minutes. Total cell proteins lysate were separated by SDS-PAGE and analyzed by Western blotting for IκB degradation. Images were processed by slight contrast adjustment using Image Lab 5.2.1 software and cropped to focus on protein of interest. (**B**) HeLa cells (4 × 10^4^ cells) stably transfected with a plasmid coding for *β-galactosidase* gene under the control NF-κB inducible promotor were co-cultured with the same amount of HeLa cells expressing X4-gp120 of HIV-1 Lai isolate. After 20 hours of co-culture, *β-galactosidase* activity was evaluated by adding the substrate of X-gal. Blue cells corresponding to *β-galactosidase* positive cells were quantified by scoring under light microscopy. The inset figure showed the nuclear translocation of NF-κB P65 subunit after 30 and 120 minutes of co-culture as described above. (**C**) Monocytes were incubated with HIV-1 gp120 (10 nM) in presence or absence of NF-κB inhibitors Bay117082 or TLCK. After 24 hours, cell supernatants were harvested and quantified by ELISA for the presence of TNF-α and IL-10. Statistical significances were compared to gp120 treated cells in the absence of inhibitor.

Considering the essential role of transcription factor NF-κB in the activation of cytokine/chemokine gene expression, including *TNF-α* and *IL-10* genes, its implication in the control of gp120-induced cytokines was evaluated by using two complementary approaches. In the first approach, we showed the capacity of gp120 to: i) activate NF-κB as demonstrated by the observed degradation of IκB in cell lysates from monocytes pre-treated during 5 to 60 min by gp120 (10 nM) (Fig. 5A, full-length blots are presented in Fig. S1) and ii) to activate the *β-galactosidase* gene expression placed on the control of NF-κB promoter (Fig. 5B). This NF-κB activation is in agreement with the nuclear translocation of P65 sub-unit of NF-κB in gp120 stimulated cells as showed in Fig. 5 B-inset (full-length blots are presented in Fig. S2). In the second approach, the involvement of NF-κB in gp120 induced TNF-α and IL-10 was evaluated by using two chemical inhibitors: TLCK, a serine/threonine protease inhibitor that inhibits NF-κB by blocking IκB degradation, and Bay-117082, an inhibitor of cytokine-induced IκB-α phosphorylation. In these conditions, the gp120-induced TNF-α and IL-10 were completely abrogated by non-cytotoxic concentrations of TLCK (200 µM) and Bay-117082 (100 µM) (Fig. 5C). Together, these results show that: *(i)* cell membrane or soluble gp120, by interacting with a potential cell surface receptor, activates the NF-κB pathway leading to TNF-α and IL-10 production; *(ii)* inhibition of NF-κB pathway abolishes gp120-induced TNF-α and IL-10; and *(iii)* gp120-induced TNF-α seems to be less sensitive to the effects of NF-κB inhibitors, TLCK and Bay-117082, when used at 20 µM and 10 µM respectively.

## Discussion

In this study, we have demonstrated that HIV-1 gp120 from either X4 or R5-tropic viruses are able to induce the production of TNF-α and IL-10 by human monocytes. However, only the analysis of the panel of signaling pathways it activates will reveal whether they activate common or specific pathways depending on their tropism. This proposal may be also extended to a future study, integrating not only the tropism (R5, X4, R5/X4, R5^+^/X4^−^, R5/X4^+^) of the envelope glycoproteins but also its viral origin (subtypes, clades and circulating recombinant forms). The amount of cytokines produced by gp120-treated monocytes appears to vary depending on both the glycoprotein preparation and the cell donors. Variations in cytokine production generated by the different used gp120 preparations can be related to intrinsic structural and/or post-translational modifications of the gp120 according to the cell system and the methods of protein production and purification. For example, it is well known that glycoprotein produced in mammalian cells or insect cells does not exhibit the same profile of glycosylation. Insect-cell-based expression systems will add shorter N-glycans, with little sialylation^58^. In line with this statement, our results show that the denaturation of gp120 by heat-treatment or modification of its secondary structure after reduction of disulfide bridges is accompanied by the abolition of its capacity to induce the production of TNF-α and IL-10. In addition to this first level of variation in the anti-gp120 response, we also observed a second level of variation that was cell donor dependent. The latter variations may be related to genetic and/or epigenetic factors related to each donor as observed in several other studies^22,59^. Such variations could also be explained by the emerging concept of “trained immunity”^60^. It is of interest to note that, in contrast to IL-1β and IL-18, which are expressed as immature pro-forms sequestered in the cytoplasm and are secreted upon catalytic cleavage by Caspase 1^61^, the production of TNF-α and IL-10 following gp120 stimulation requires *de-novo* protein synthesis, as demonstrated by the capacity of cycloheximide, an inhibitor of protein synthesis, to block TNF-α and IL-10 secretion.

Given that T cells are also able to produce IL-10 and TNF-α, we asked whether they participated in the production of cytokines following stimulation with gp120. To answer this question, we tested the ability of gp120 to induce the production of TNF-α and IL-10 in the lymphocyte fraction of PBMC, enriched by elimination of adherent cells after three successive adherence steps. In these conditions, no significant cytokine production was observed in cell supernatants of the monocyte-free non-adherent cells treated with gp120, thus indicating the direct implication of monocytes.

Several other studies have shown that, in addition to TNF-α and IL-10, HIV-1 enveloped glycoproteins also stimulate the production of a variety of pro-inflammatory and anti-inflammatory cytokines/chemokines, including IL-1β, IL-6, IL-8, IL-18, IL-23, IL-27, CCL2, CCL4, CCL20, CXCL2, CXCL13, and TSLP^62–64^ that are also implicated in HIV-1 associated impairment of the immune system^52,53,63^, AIDS-related diseases, and the aberrant sustained hyper immune-activation^65,66^.

Therefore, new targets and therapeutic approaches that control TNF-α and IL-10 production could be helpful to curtail hyper immune activation and T cell exhaustion in HIV-infected patients. The relationship between chronic immune activation and AIDS-disease progression has been studied extensively in both human and non-human primate models. In the human model, one striking hallmark of elite controllers is the absence of hyper-immune activation. A similar observation has been made in the SIV/animal model. The administration of the pathogenic SIVmac251 or SIVmac239 strain^67^ to Sooty-Mangabey or African green monkeys, the natural hosts of SIV, leads to infection with a high viral load but the animals do not develop an AIDS-like disease. In contrast, the same experimental infection of Asian macaques, a non-natural host of SIV, leads to their infection and AIDS disease development. The major difference between these two models is that the natural host does not develop persistent hyper immune-activation, while the non-natural host develops persistent chronic hyper-immune-activation. These two key observations in HIV-1 elite controllers and in host and non-host monkeys underline the importance of immune activation in disease development and thus, justify further investigations in order to understand the molecular and cellular mechanisms governing these aberrant immune-hyper activations.

The findings of the present study are in contrast with the published data from other groups showing that gp120-induced cytokines are dependent on CD4 receptor^7,68,69^, or independent of CD4 but dependent on CCR5/CXCR4 co-receptors^70^. Here, we show that stimulation of TNF-α and IL-10 production by X4-tropic gp120 seems to be independent of both CD4 and CXCR4, as demonstrated by the incapacity of blocking anti-CD4 antibodies, soluble CD4 and AMD300, a known antagonist ligand of CXCR4, to inhibit gp120-induced cytokines. These results suggest the implication of another receptor for gp120. HIV-1 gp120 is a highly glycosylated protein; about 50% of its molecular mass is composed of carbohydrates^71^. In addition to CD4 and CCR5/CXCR4, HIV-1 gp120 has been reported to engage several mannose C-type lectin receptors (MCLR) including mannose receptor (MR)^22^. In our study, no inhibition of gp120-induced cytokines was observed in the presence of mAb 2G12, an HIV-1 neutralizing antibody directed against mannose motifs on gp120, while other studies showed only a partial inhibition of cytokine-induced gp120 with this mAb, anti-DC-SIGN mAb AZN-D1 or Cyanovirin, a bacterial protein displaying a high affinity to high mannose oligosaccharides^22^. In line with our data, other works have shown that gp120 is also able to induce IL-10 production in mice^72^ despite the fact that gp120 is unable to bind murine CD4, CXCR4, CCR5 or MCLR^72,73^. Altogether, these data suggest that gp120 engages a receptor, different from CD4 and CCR5/CXCR4 and DC-SIGN, which remains to be defined. Other receptors have been described for gp120, including different MCLR, α4β7 integrin^74^, TLR2^75,76^ TLR4^41^or both receptors^42^ that could mediate the production of various cytokines/chemokines in addition to TNF-α and IL-10.

The engagement of cell surface receptors by gp120 is followed by the activation of multiple cell signaling pathways, leading to the positive or negative modulation of different genes. Accordingly, the analysis by oligonucleotide microarray of the quiescent CD4 T cells isolated from non-infected patients, activated by the envelope glycoproteins gp120 from X4 or R5-tropic viruses, revealed the selective positive modulation of 587 genes, and 822 genes respectively. 699 genes were activated by both R5 and X4 gp120^77^. Among all the genes that were up-regulated, there were genes associated with cell proliferation, cell cycle, and transcription factors^77^. In a more quantitative phosphoproteomic study, it was shown that the stimulation of quiescent primary CD4 T cells for 1 minute with X4-tropic HIV-1 allowed the phosporylation of 175 genes, many of which were involved in the steps of viral entry, actin cytoskeleton rearrangement, reverse transcriptase activity, movement of the complex of reverse transcription into the center of the cytoplasm, nuclear translocation and integration into the genome of the host cell^78^.

The calcium pathway is known for its importance among the signaling pathways controlling the expression of cytokine/chemokine genes, so we tested its role in the induction of TNF-α and IL-10 by human monocytes following their stimulation by HIV-1 gp120. Our findings show, for the first time, that: *(i)* when the stimulation of monocytes was performed in the absence of calcium, the IL-10 production was totally blocked, while the monocytes continued to produce significant amounts, about 42%, of TNF-α. Interestingly, IL-10 blockade can be completely rescued by the addition of exogenous Ca^2+^; *(ii)* total inhibition of IL-10, was obtained in the presence of BAPTA-AM, a chelator of intracellular calcium, while no effect was observed on the production of TNF-α; *(iii)* inhibition, by cyclosporine-A of calcineurin, a phosphatase responsible for the activation of the transcription factor NFAT, leads to strong inhibition of IL-10, without affecting TNF-α production; and *(iv)* in agreement with the above three points, inhibition of PKA, a Ser/Thr kinase that has been reported for its involvement in the phosphorylation of calcium channels, including Ryanodine receptors and L-type calcium channels^79,80^, leads to the inhibition of gp120-induced IL-10 production.

Altogether, our findings underline the role of the calcium pathway in the control of IL-10 production and suggest the involvement of an alternative, calcium independent, pathway in the control of TNF-α production by gp120-stimulated monocytes. This crucial role of calcium pathway is in line with previous reports describing the capacity of gp120 to induce calcium mobilization^81^. It is interesting to note that this dependence on calcium was inverted in the HIV-1 Tat-monocyte/model previously described by our group^18,82–85^. In this model, in contrast to the mode of action of gp120, the absence of calcium is found to block TNF-α completely, while IL-10 production in Tat-treated monocytes was partially blocked^86^. In addition, in this model, it has been observed that PKC, but not PKA nor MAP kinases ERK1/2, is required for Tat-induced TNF-α and IL-10 production^18,82,87^. Moreover, an inverted involvement was found, where PKA and MAP kinases ERK1/2, but not PKC, pathways seemed to be essential for the production of TNF-α and IL-10 by gp120-stimulated monocytes. These reports are one illustration of the capacity of HIV-1 to hijack different host cell pathways to positively modulate the production of TNF-α and IL-10, two cytokines highly implicated both in the chronic activation and the weakness of the immune system. In addition, our findings also underlined the crucial importance of NF-κB pathway in gp120-induced TNF-α and IL-10 cytokines.

**Figure 6:**
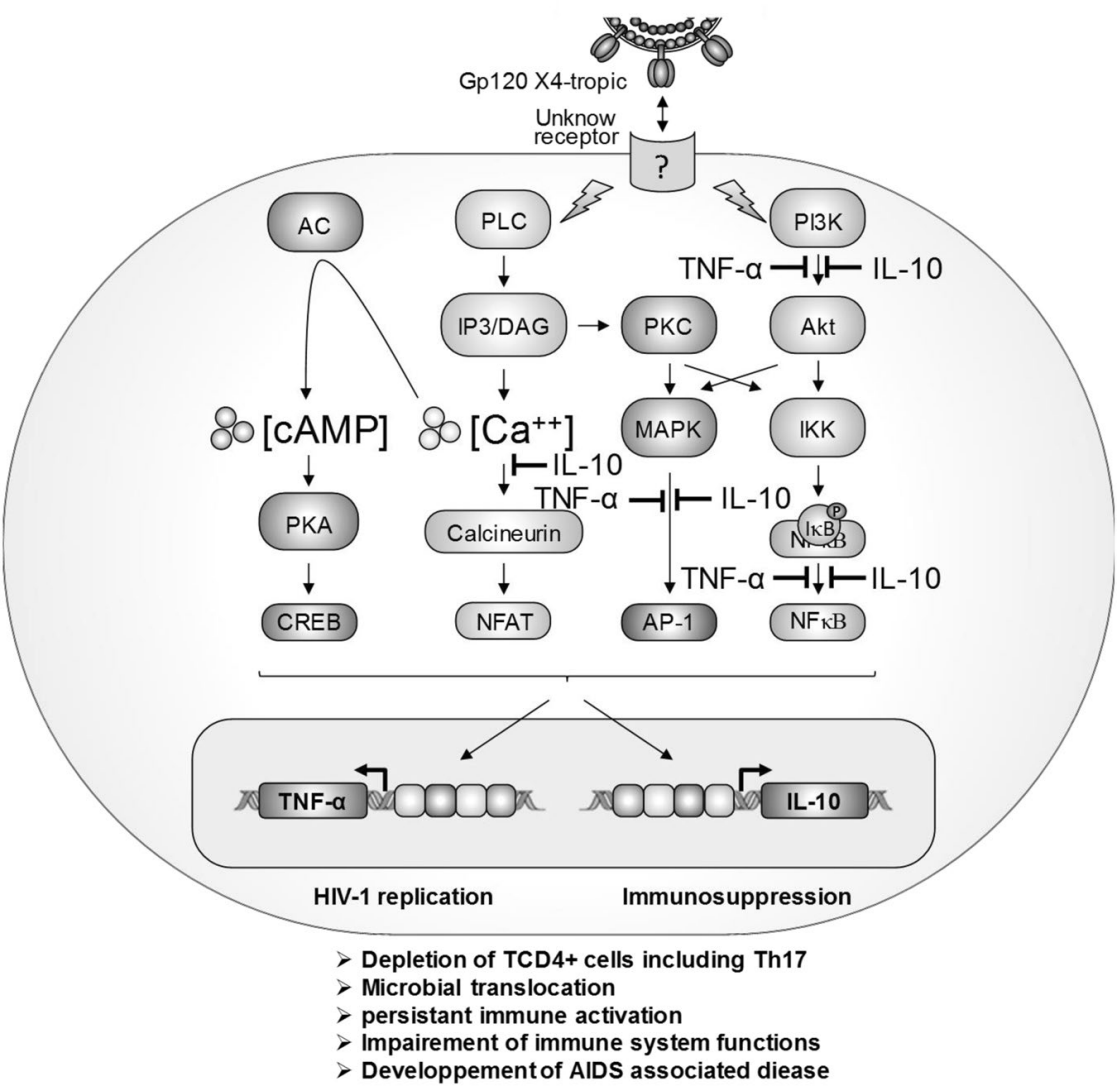
Model of X4-gp120 activated signaling pathways involved in the activation of TNF-α and IL-10 gene expression. Two important points can be highlighted from this model: First, the key receptor, which remains to be defined, involved in the induction of TNF-α and IL-10 gene expression is different from CD4, CXCR4 and MR. Second, X4-gp120 continues to signal through these historical receptors to activate specific or amplify common pathways with the yet undefined receptor to activate or enhance the production of these two immune-regulatory cytokines: TNF-α and IL-10. The crucial pathways controlling 100% of TNF-α or IL-10 production are depicted by the symbol .

In conclusion, our results show that X4-tropic gp120 induced TNF-α and IL-10 production by monocytes through activation of yet-to-be-determined receptor(s), different from CD4, CXCR4 and MR. The analysis of signaling pathways required for gp120-induced TNF-α and IL-10 production showed that both cytokines are dependent on PLC, PI3K, ERK1/2, p38 MAP kinases and NF-κB pathways. While PKA and calcium pathways are essential for gp120-induced IL-10 production, they are only partially involved in gp120-induced TNF-α production (Fig. 6). This work describes essential signaling pathways triggered by HIV-1 gp120 to induce the expression of immune-regulatory cytokines TNF-α and IL-10. The finding could help in the development of new therapeutic strategies to alleviate immune activation in restoring the immune function during HIV-1 infection.

## Electronic supplementary material

Supplementary Figures

## Data Availability

The datasets generated during and/or analysed during the current study are available from the corresponding author on reasonable request.
