# Supplementary material for: HIV-1 Envelope Glycoproteins Induce the Production of TNF-α and IL-10 in Human Monocytes by Activating Calcium Pathway
Source: Sci Rep. 2018 Nov 21;8:17215. doi: 10.1038/s41598-018-35478-1 (PMC6249280; doi:10.1038/s41598-018-35478-1)
Supplement: Supplementary file 1 — Supplementary Figures [file 41598_2018_35478_MOESM1_ESM.pdf]

HIV-1 Envelope Glycoproteins Induce the Production of TNF-α and IL-10 in Human Monocytes by Activating Calcium Pathway.

Rémi Planès[1, 2, 3]; Manutea Serrero[1, 2, 3]; Kaoutar Leghmari[1, 2, 3]; Lbachir BenMohamed[4]

& Elmostafa Bahraoui[1, 2, 3, *]

Supplementary ionformations

Supplementary Figure 1

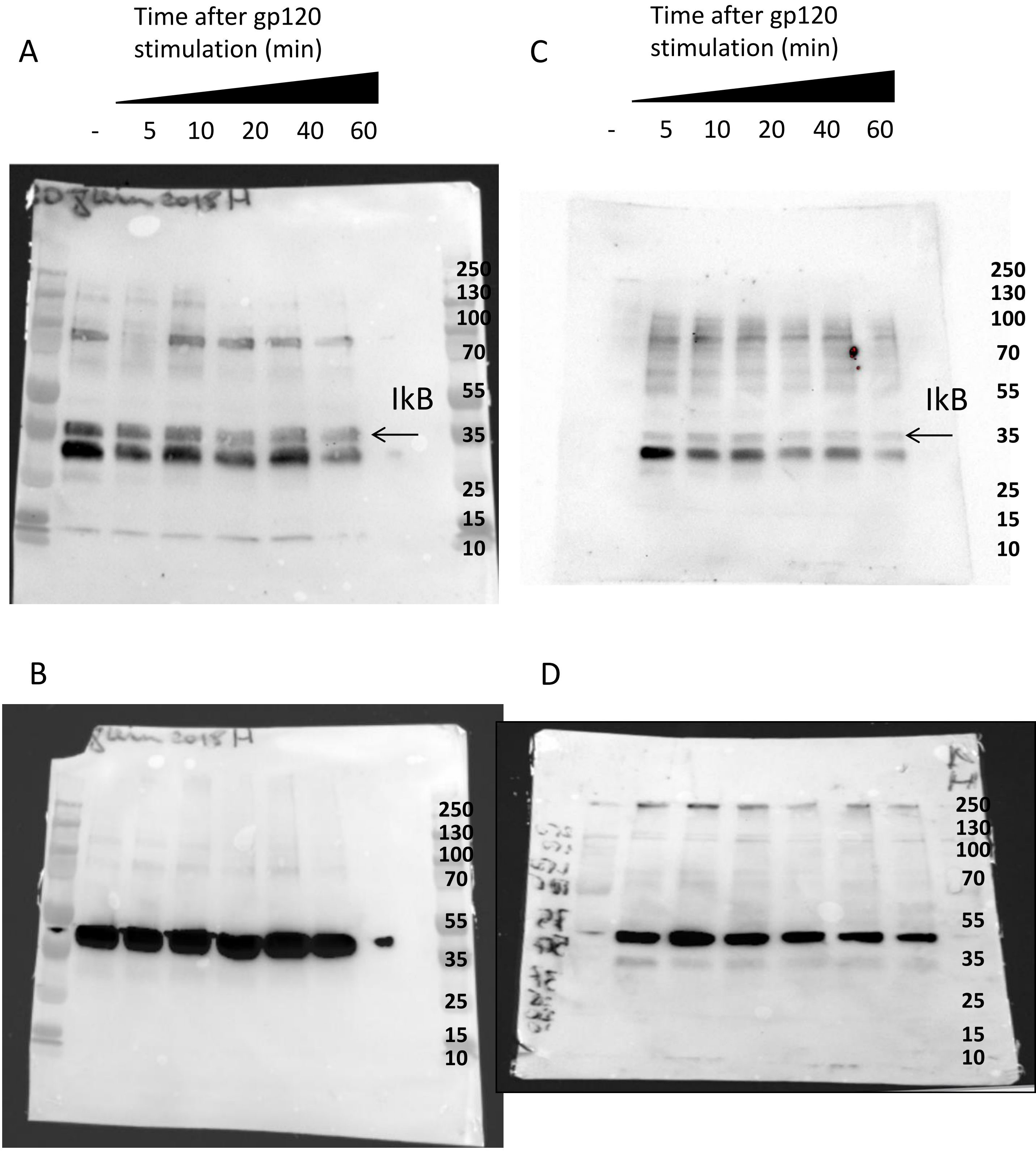

Figure S1.
(**A-B**) Original blot images unprocessed related to figure 5A presented in the manuscript. (**A, C**) show IκB staining (**B, D**) show Actin staining. Two independent experiments are shown.

Supplementary Figure 2

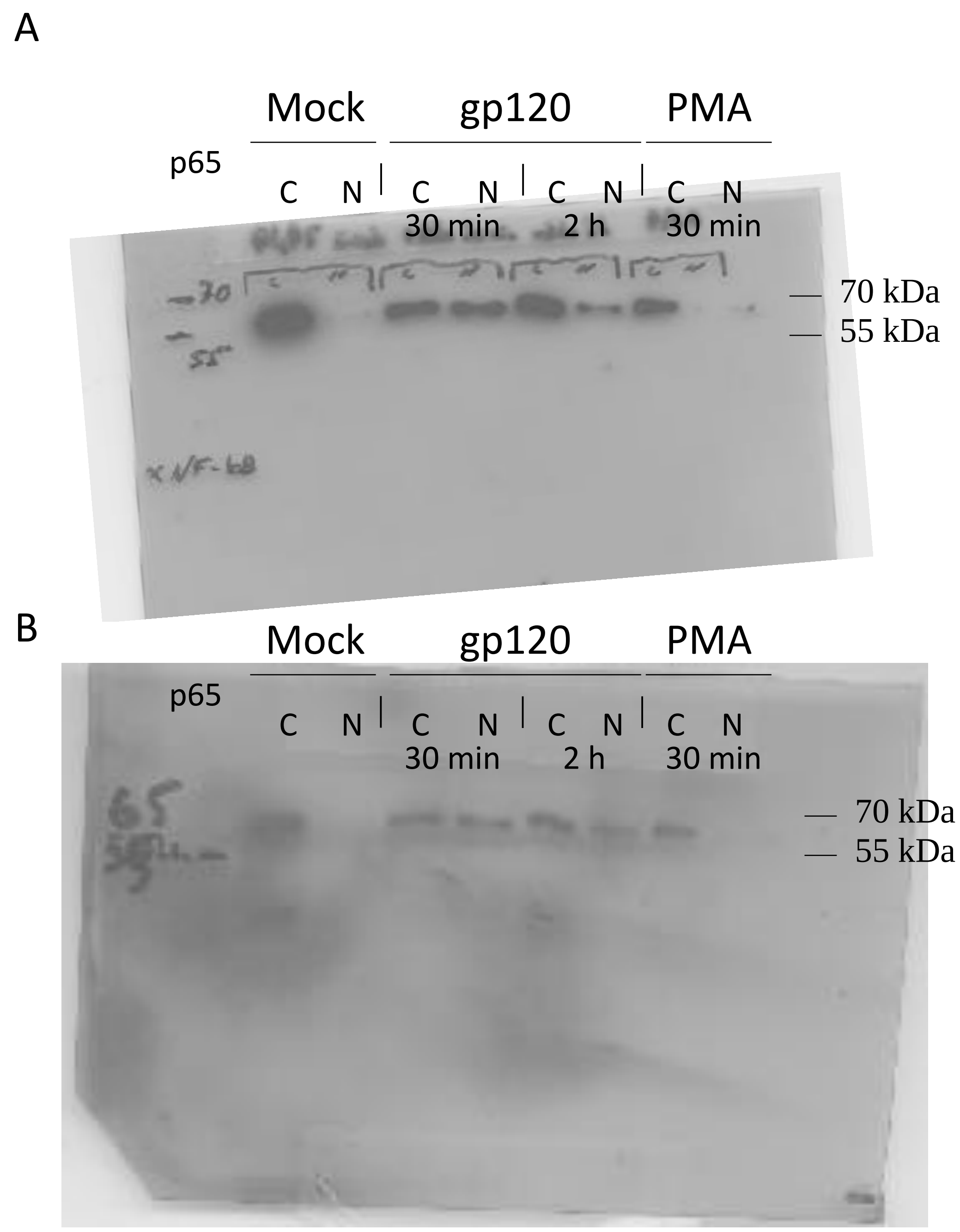

Figure S2.
(**A**) Original blot images unprocessed related to figure 5B presented in the manuscript. (**A-B**) show NF-κB p65 staining from either cytoplasmic (C) or nuclear (N) fractions. Two independent experiments are shown.
